# Social Motor Coordination in Unaffected Relatives of Schizophrenia Patients: A Potential Intermediate Phenotype

**DOI:** 10.3389/fnbeh.2013.00137

**Published:** 2013-10-02

**Authors:** Jonathan Del-Monte, Delphine Capdevielle, Manuel Varlet, Ludovic Marin, Richard C. Schmidt, Robin N. Salesse, Benoît G. Bardy, Jean Philippe Boulenger, Marie Christine Gély-Nargeot, Jérôme Attal, Stéphane Raffard

**Affiliations:** ^1^Epsylon, Laboratory Dynamic of Human Abilities and Health Behaviors, Department of Sport Sciences, Psychology and Medicine, University of Montpellier, Montpellier, France; ^2^Movement to Health Laboratory, EuroMov, Montpellier-1 University, Montpellier, France; ^3^Department of Adult Psychiatry, CHU Montpellier, Hôpital de la Colombière, Montpellier-1 University, Montpellier, France; ^4^U-1061, INSERM, Montpellier, France; ^5^Department of Psychology, College of the Holy Cross, Worcester, MA, USA; ^6^Institut Universitaire de France, Paris, France; ^7^Epsylon, Laboratory Dynamic of Human Abilities and Health Behaviors, Department of Sport Sciences, Psychology and Medicine, University of Montpellier, Montpellier, France

**Keywords:** unaffected first-degree relatives, schizophrenia, interpersonal motor coordination, intermediate phenotypes, social interaction

## Abstract

Intermediate endophenotypes emerge as an important concept in the study of schizophrenia. Although research on phenotypes mainly investigated cognitive, metabolic or neurophysiological markers so far, some authors also examined the motor behavior anomalies as a potential trait-marker of the disease. However, no research has investigated social motor coordination despite the possible importance of its anomalies in schizophrenia. The aim of this study was thus to determine whether coordination modifications previously demonstrated in schizophrenia are trait-markers that might be associated with the risk for this pathology. Interpersonal motor coordination in 27 unaffected first-degree relatives of schizophrenia patients and 27 healthy controls was assessed using a hand-held pendulum task to examine the presence of interpersonal coordination impairments in individuals at risk for the disorder. Measures of neurologic soft signs, clinical variables and neurocognitive functions were collected to assess the cognitive and clinical correlates of social coordination impairments in at-risk relatives. After controlling for potential confounding variables, unaffected relatives of schizophrenia patients had impaired intentional interpersonal coordination compared to healthy controls while unintentional interpersonal coordination was preserved. More specifically, in intentional coordination, the unaffected relatives of schizophrenia patients exhibited coordination patterns that had greater variability and in which relatives did not lead the coordination. These results show that unaffected relatives of schizophrenia patients, like the patients themselves, also present deficits in intentional interpersonal coordination. For the first time, these results suggest that intentional interpersonal coordination impairments might be a potential motor intermediate endophenotype of schizophrenia opening new perspectives for early diagnosis.

## Introduction

Schizophrenia is a highly heritable condition, with complex genetic susceptibility likely arising from the combined effects of multiple susceptibility alleles of individually weak effect, and environmental factors. This complicates the search for susceptibility genes with traditional linkage approaches (Gottesman and Shields, 1973). An alternative approach is based on the identification of so-called intermediate phenotypes that are detectable both in schizophrenia patients and in a higher proportion of their unaffected relatives than in the population at large (Pearlson and Folley, 2008). The advantage of such intermediate phenotypes stems from their greater penetrance at the level of vulnerability markers, thus increasing statistical power (Egan et al., 2001). A more recently employed strategy is thus to study unaffected relatives of schizophrenia patients, who share some of the genetic diathesis without illness-related confounds (e.g., medication effects) that may themselves impact task performance. Researchers have proposed several criteria, for useful intermediate phenotypes with regard to schizophrenia and other psychiatric disorders (Gottesman and Gould, 2003; Gould and Gottesman, 2006). Although there is no universally agreed-upon definition or evaluation of a promising intermediate phenotype, all share, and highlight several key elements in the inclusion criteria (Glahn et al., 2007). To be useful an intermediate phenotype must be: (1) associated with the illness; (2) heritable to some degree; (3) observed in unaffected family members to a greater extent than the general population, and (4) state independent in affected individuals (Gottesman and Gould, 2003). Recent research focusing on schizophrenia has led to candidate variables as intermediate phenotype markers (B-SNIP, Thaker, 2007) for the illness.

Although research on intermediate phenotypes mainly investigated cognitive (Snizt et al., 2006), emotional (Erol et al., 2010), or neurobiological (Woodward et al., 2009) markers so far, some authors also examined motor behavior anomalies as potential phenotypes in schizophrenia. The motor abnormalities most studied in relatives of schizophrenia patients have been neurological soft signs (NSS), including difficulties with sequencing of motor tasks, and subtly impaired sensory integration (Neelam et al., 2011). NSS are observable in first-episode patients (Bachmann et al., 2005), and even prior to antipsychotic administration (Scheffer, 2004; Chen et al., 2005). Additionally, adolescents who later develop schizophrenia exhibit signs of neurological abnormalities, particularly motor dyscoordination, well before overt manifestations of the illness (Leask et al., 2002; Mittal et al., 2011), providing further evidence for a fundamental role of motor dysfunctions as an early constitutional vulnerability of schizophrenia. Several studies showed that NSS are present in relatives of schizophrenia patients (Chan et al., 2010; Neelam et al., 2011), and are among the most promising of the candidate intermediate phenotypes (Chan and Gottesman, 2008). Whereas these studies only examined intrapersonal motor behaviors, other studies have found interpersonal motor coordination (IMC) is also impaired in schizophrenia (Varlet et al., 2012b). Interpersonal coordination (e.g., the synchronization of movements between two individuals that emerged during a social interaction) plays an important role in everyday life (Schmidt et al., 2011). For example, the effectiveness of an interaction can often be directly linked to the degree of interpersonal coordination (Marsh et al., 2009), and consequently, the social motor anomalies shown in patients with schizophrenia might play a crucial role in their interpersonal deficits that are frequently reported in the literature.

Early studies on human motor coordination were initially interested in the intrapersonal motor coordination, such as rhythmic bimanual motor coordination tasks (i.e., the coordination of the two hands or fingers of the same person) (Kelso, 1984; Kelso and Schöner, 1988; Schmidt et al., 1993; Peper et al., 1995; Riley et al., 2001). These studies showed that two patterns of stability were preferentially adopted, in-phase and anti-phase patterns, respectively characterized by movements in the same and opposite directions (i.e., relative phase value of 0° and 180°) and that in-phase movements were more stable than anti-phase movements. The exact same results were found in IMC studies (e.g., Schmidt et al., 1990; Amazeen et al., 1995). Schmidt et al. (1990) investigated the differential stability of different relative phase patterns in between-person coordination of lower leg oscillations. They found that, as in intrapersonal coordination task, in-phase movements were more stable than anti-phase movements. Because similar phenomenon had been observed in the within-person coordination of hand movements (Kelso, 1984) and have been dynamically explained using a coupled oscillator model (Haken et al., 1985; Schöner et al., 1986), Schmidt et al. (1990) argued that the interpersonal differential stability of relative phase patterns has an identical dynamical explanation. Additional evidence for a dynamical organizing of interpersonal coordination was found using a wrist-pendulum methodology. Schmidt and Turvey (1994) and Amazeen et al. (1995) had two participants sitting side-by-side coordinate the oscillation of weighted dowels – wrist pendulums – in the sagittal plane using ulnar and radial flexion of their wrists. Their task was to watch each other’s pendulum and to coordinate their oscillations. The results of the between-person wrist-pendulum experiment provided clear support for the dynamical, coupled oscillator model: as the eigenfrequencies (inherent, preferred frequencies) of the pendulum pairs became more different, the mean relative phase of the system moved from perfectly in-phase or anti-phase such that the pendulum with the inherently lower eigenfrequency always lagged in its cycle, and the standard deviation of relative phase increased. These studies suggested that interpersonal coordination could be understood in terms of the same dynamical processes of self-organization that was used to understand intrapersonal coordination.

Classical interpersonal coordination studies not only investigated intentional interpersonal synchronization but also unintentional or spontaneous interpersonal synchronization as well. In interaction situations in which two subjects are moving rhythmically, as soon as visual information is exchanged between the interacting people (van Ulzen et al., 2008), their movements spontaneously and intermittently synchronize toward the in-phase and anti-phase patterns of coordination (Schmidt and O’Brien, 1997; Richardson et al., 2007). These studies suggest that implicit interpersonal synchronization although weaker than explicit intentional interpersonal synchronization occurs outside of people’s awareness and potentially operates in the social motor behavior underlying everyday interactions (Schmidt et al., 2011).

In Varlet et al.’s (2012b) study, schizophrenia patients and healthy controls were asked to perform an IMC task using the same hand-held pendulums methodology (Schmidt and Turvey, 1994; Schmidt and O’Brien, 1997). The goal of this study was to evaluate the dynamics of IMC in people affected by schizophrenia in order to further characterize their abnormal interpersonal movements. The results showed that schizophrenia patients had unimpaired unintentional interpersonal motor coordination (UIMC) but impaired intentional interpersonal motor coordination (IIMC) compared to healthy controls. The abnormalities found for intentional coordination were never before observed in the literature concerning schizophrenia. These abnormalities are characterized by IIMC more variable than controls and with patients never leading the coordination, suggesting that they originate from a decrease in the strength of the coupling as well as a delay in the information transmitted about movements of the other person (Varlet et al., 2012b).

**Figure 1:**
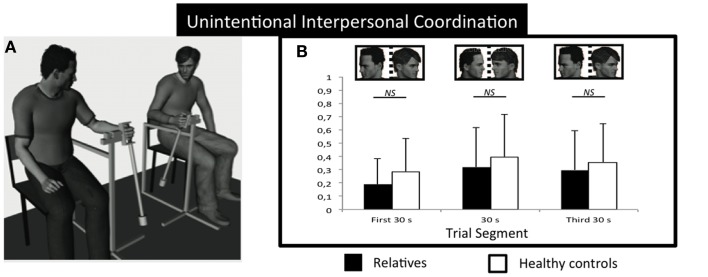
**Experimental setup and results for unintentional social motor coordination**. **(A)** Participants sat on chairs side- by-side while oscillating wrist pendulums. Circular variance of the relative phase for the relatives (black) and control groups (white) as a function of the trial segment for unintentional coordination **(B)**. No significant differences between groups. NS, not significant. Error bars represent standard error.

Thus, IMC assessment seems to open a new perspective on understanding the social disorders in mental illness and, consequently, research should take into account interpersonal motor markers in these disorders (Varlet et al., 2012b). Accordingly, the aim of the present study was to determine whether or not the motor coordination abnormalities found in schizophrenia patients, could be a potential trait-marker for schizophrenia. To accomplish this, we evaluated the dynamics of UIMC and IIMC of unaffected relatives of schizophrenia while swinging hand-held pendulums with another individual (see Figure 1A). We compared the motor coordination produced by relatives of patients paired with healthy participants (relatives group) and the coordination produced by pairs of healthy control participants (control group). As suggested by Varlet et al. (2012b), it was predicted that if there were a potential trait-marker for schizophrenia, relatives of schizophrenia patients would present IMC deficits characterized by more variable IIMC than controls and with relatives never leading the coordination. Additionally, we also assessed the potential relationships between IMC performance and cognitive and clinical variables in unaffected relatives of schizophrenia patients.

## Materials and Methods

### Participants

Twenty-seven relatives (14 mothers and 13 fathers) of schizophrenia patients were recruited through the University Department of Adult Psychiatry in Montpellier, France. Another 27 participants healthy matched control participants were also recruited. To create dyads for testing social coordination, the 27 first-degree relative participants and the 27 matched control participants were randomly paired with 54 unmatched healthy partners. All partner pairs were of the same sex to avoid methodological bias (Katic et al., 2012, 2013; Johansen et al., 2013). But, note that although 54 healthy partners were unmatched on the age, level education, and premorbid IQ with the relatives and controls, they were matched between them (see Table 1). Consequently, a total of 108 participants were combined into 54 pairs (27 pairs in relatives group and 27 pairs in control group) for this study.

**Table 1:** **Mean ± SD of demographic characteristics of participants**.

	Matching 1				Matching 2			
	Relatives (*n* = 27)	Controls (*n* = 27)	Statistics	*p*	Partner 1 (*n* = 27)	Partner 2 (*n* = 27)	Statistics	*p*
Age (years)	61.37 ± 6.48	59.74 ± 6.99	*z* = −1.196^a^	0.16	24.70 ± 5.89	26.66 ± 10.51	z = −0.600^a^	0.54
Sex (male/female)	13/14	13/14	χ^2^ = 0^b^	1	13/14	13/14	χ^2^ = 0^b^	1
Education (years)	14.18 ± 3.39	13.44 ± 1.84	*z* = −0.814^a^	0.41	15.22 ± 2.37	14.55 ± 2.30	z = −0.798^a^	0.42
Premorbid IQ (*f*NART)	110.18 ± 7.57	113.22 ± 6.03	*z* = −1.242^a^	0.16	112.85 ± 4.51	109.88 ± 6.28	z = −1.513^a^	0.13

IQ, intellectual quotient; fNART, French version of the National Adult Reading Test,

^a^U-Mann–Whitney non-parametric test,

^b^Chi-square test.

All control participants and healthy partners were community-dwelling adults living in Montpellier, and were recruited from local associative networks. Exclusion criteria for the relatives, controls, and partners were positive history of neurological or psychiatric disease or the presence of medication intake known to influence cognition and movement function. Thus, participants receiving antipsychotic, antidepressant drugs, or benzodiazepines that might influence motor behavior or cognitive functioning were excluded. Control participants, meeting clinical criteria for major depressive episode or anxiety disorder as confirmed using the mini-international neuropsychiatric interview (Sheehan et al., 1998) were also excluded. All participants had normal or corrected-to-normal vision. They provided written informed consent prior the experiment approved by the local Ethics Committee (CPP Sud Méditérannée III, Montpellier, France, AFSSAPS 2009-A00513-54 24, 07/22/2009) conforming to the Declaration of Helsinki.

### Materials

Subjects were asked to complete questionnaires and to participate in an IMC task. A psychiatrist experimenter blind to the hypotheses assessed all subjects. The total experiment time was approximately 2 h for a pair of participants. For all subjects, we assessed, global cognitive functioning using the Mini Mental State Examination (Folstein et al., 1975), NSS (Krebs et al., 2000), premorbid IQ using the French National Adult Reading Test (Mackinnon and Mulligan, 2005), depression using the Beck Depression Inventory-II (Beck et al., 1996), and social anxiety using the Liebowitz-Social-Anxiety-Scale (Liebowitz, 1987). Additionally, for all subjects, visual sustained attentional function was assessed using the Concentration-Endurance Test (Brickenkamp, 1994). In this test, items are composed of the letters “d” and “p” with one, two, three, or four dashes arranged either individually or in pairs above and below the letter. Participants are given 20 s to scan each line and mark all “d’s” with two dashes.

### Interpersonal motor coordination task

We examined the IMC of unaffected relatives while coordinating the swinging of hand-held pendulums with another individual (Amazeen et al., 1995; Schmidt and O’Brien, 1997) (see Figure 1A).

Participants sat on chairs approximately 1 m from one another facing in the same direction and swung wrist pendulums attached to a structure that allowed only movements from front to back (see Figure 1A). The swinging of the pendulums made no noise that could be used as a cue for coordination. The length of the two pendulums was 60 cm and a mass of 150 g was attached either at the bottom or at the middle to respectively compose the pendulums of different inertias which would manipulate the self-selected comfort tempo (preferred frequencies) of participants. For those pendulums that had greater inertia, and hence slower comfort tempos, past research found that they would lag their partner’s pendulum throughout the cycle whereas pendulums with less inertia were found to lead their partner’s. Consequently the individual pendulums will be referred to as FOLLOWER and LEADER, respectively. Two computer-monitored potentiometers measured the angular displacements of the pendulums during the trials at a sampling rate of 50 Hz.

### Procedure

Participants were first tested for UIMC. They performed six trials of 90 s for each of the following pendulum combinations: LEADER_FOLLOWER, FOLLOWER_FOLLOWER, and FOLLOWER_LEADER where the first pendulum of the pair refers to the pendulum used by relative group participant or the matched control group participant and the second pendulum refers to the pendulum used by their partners. Each trial contained three 30 s segments that were delimited by an auditory stimulus. During the first and third segments, participants were instructed to focus on crosses that were positioned on the wall in the opposite direction of the other participant, which allowed indexing chance level coordination. During the second segment, they were instructed to look at the movements of their partner. Participants were instructed to swing the pendulum at their own self-selected tempo and to maintain that tempo throughout the trial, irrespective of whether they were looking (second segment) or not looking (first and third segments) at the movements of their partner (Schmidt and O’Brien, 1997). The order of the pendulum combinations was counterbalanced across participant pairs.

Participant pairs were then tested for intentional IMC. They were instructed that they would be required to coordinate their movements together in an in-phase or anti-phase mode for 60 s (Amazeen et al., 1995; Richardson et al., 2007). After the instructions, each pair of participants performed one practice trial for each of the experimental conditions. They then performed six trials for each pattern of coordination with the three different pendulum combinations (LEADER_FOLLOWER, FOLLOWER_FOLLOWER, and FOLLOWER_LEADER). The order of the trials was counterbalanced across participants.

### Statistical analysis

Clinical ratings and cognitive performances were separately compared for the two groups with non-parametric *U*-Mann–Whitney tests. The level of significance was set to *p* < 0.05. Spearman non-parametric correlations were used to explore the relationships between IMC, cognitive, and clinical variables.

Concerning the coordination analysis, the first 5 s of each intentional trial and each segment of unintentional trials were discarded to avoid transient behavior as in Varlet et al.’s (2012b) study. The time series of participants were low-pass filtered using a 10 Hz bidirectional Butterworth filter, a signal-processing filter typically employed in human movement research to remove noise from movement time series (Winter, 1990). We used this filter in the current study although it was almost unnecessary because the data collected were not noisy. The continuous relative phase between the two angular positions of the pendulums was computed using the Hilbert Transform (Pikovsky et al., 2003). For both unintentional and intentional trials, we calculated the circular variance of the relative phase to provide an index of synchronization between 0 (no synchronization) and 1 (perfect synchronization). For intentional coordination trials, we also computed the phase shift to further explore the stability of the coordination and determine which participant led the coordination. As mentioned above, the Leadership of the coordination depends also on the pendulums used by participants and can be explained in terms of the difference between the pendulums’ eigenfrequencies. Past studies (Amazeen et al., 1995; Richardson et al., 2007; Varlet et al., 2012b) have showed a phase shift of 0° when participants used the same pendulums (FOLLOWER_FOLLOWER) indicating no leader in the dyad, and a negative phase shift for LEADER_FOLLOWER and a positive phase shift for FOLLOWER_LEADER indicating the Leadership of the participant who used the fast pendulum (LEADER). Positive phase shifts indicated that relatives or control group participants led the coordination whereas negative phase shifts indicated that relatives or control group participants followed the movements of the other participant. We used analyses of variance (ANOVAs) to examine the different variables and Bonferroni corrected *post hoc* tests to determine the nature of the effects when necessary. Single sample *t*-tests were also used to determine whether phase shift values were significantly different from zero.

## Results

Mean ratings and group comparisons on the demographic data are reported in Table 1. Mean ratings and group comparisons on the clinical and cognitive variables are reported in Table 2. The results revealed that unaffected relatives have less sustained visual attentional (*z* = −2.804, *p* = 0.005, $\eta_{p}^{2}=0.327$) and more NSSs (*z* = −2.370, *p* = 0.018, $\eta_{p}^{2}=0.384$) compared to healthy controls.

**Table 2:** **Means ± SD of clinical characteristics of participants**.

Variables	Relatives group (*n* = 27)	Control group (*n* = 27)	*df*	Statistics	*p*	$\eta_{p}^{2}$
Cognitive global functioning (MMSE)	28.92 ± 0.87	29.22 ± 1.05	52	*z* = −1.527^a^	0.12	0.20
Depression (BDI–II)	10 ± 9.21	9.44 ± 9.11	52	*z* = −0.026^a^	0.97	0.003
LSAS anxiety	14.18 ± 7.81	16.81 ± 11.57	52	*z* = −0.303^a^	0.76	0.041
LSAS avoidance	13.11 ± 6.25	14.59 ± 8.85	52	*z* = −0.468^a^	0.64	0.063
LSAS anxiety in social interaction	5.29 ± 4.25	6.59 ± 5.39	52	*z* = −0.772^a^	0.44	0.10
LSAS avoidance in social interaction	5.66 ± 3.22	6.03 ± 4.60	52	*z* = −0.174^a^	0.86	0.023
LSAS anxiety of performance	8.88 ± 4.49	10.22 ± 6.55	52	*z* = −0.442^a^	0.65	0.06
LSAS avoidance of performance	7.37 ± 3.98	8.18 ± 4.89	52	*z* = −0.542^a^	0.60	0.073
Visual sustained attention score (*d*_2_)	153.88 ± 38.08	177.44 ± 43.22	52	*z* = −2.804^a^	0.005**	0.38
Visual sustained attention error score (*d*_2_)	5.50 ± 5.12	5.12 ± 6.96	52	*z* = −0.770^a^	0.441	0.10
Neurologic soft signs score (NSS) total	7.68 ± 4.15	5.48 ± 3.93	52	*z* = −2.370^a^	0.018*	0.32
Motor coordination	3.69 ± 2.22	2.88 ± 2.23	52	*z* = −1.651^a^	0.099	0.22
Motor integration	0.69 ± 1.13	0.77 ± 1.11	52	*z* = −0.537	0.587	0.07
Sensory integration	2.06 ± 0.97	1 ± 1	52	*z* = −3.840	0.001**	0.52
Involuntary movement	0.13 ± 0.45	0.15 ± 0.36	52	*z* = 0	1	0
Lateralization	1.04 ± 1.34	0.66 ± 1.20	52	*z* = −1.311	0.190	0.18

*LSAS; Liebowitz-social-anxiety-scale, MMSE; mini mental state examination, BDI-II; Beck depression inventory version II*.

^a^U-Mann–Whitney non-parametric test,

^b^Chi-square test. *p < 0.05; **p < 0.01.

### Unintentional interpersonal motor coordination

For UIMC, the ANOVA on relative phase circular variance revealed significant main effects of Trial Segment (*F*(2, 25) = 9.908, *p* < 0.002, $\eta_{p}^{2}=0.442$) and of Pendulum Combination (*F*(2, 25) = 7.467, *p* < 0.005, $\eta_{p}^{2}=0.374$) (Figure 1B). As expected, *post hoc* comparisons revealed stronger synchronization when visual information was available about the partner’s movements (i.e., stronger synchronization in second segment compared to the first and third segments, *p* < 0.05) and when participant oscillated the same pendulums (i.e., stronger synchronization in FOLLOWER_FOLLOWER condition compared to FOLLOWER_LEADER and LEADER_FOLLOWER, *p* < 0.05). These results affirm previous UIMC studies that have found evidence for coupled oscillatory processes coordinating interpersonal rhythmic movements implicitly outside of inter-actors awareness (Schmidt and O’Brien, 1997). However, note that the analysis did not show any significant effect of Group on UIMC, which suggests that the implicit harnessing of such coupled oscillatory processes is not impaired in the unaffected relative group participants.

### Intentional interpersonal motor coordination

#### Phase shift of the relative phase

In previous research, the phase shift (i.e., which pendulum is leading) has been used to further explore the stability of the coordination and determine which participant led the coordination (Amazeen et al., 1995; Richardson et al., 2007). Here, positive phase shifts indicated that the unaffected relatives led the coordination and negative phase shifts indicated that unaffected relatives followed the other participant.

The ANOVA on the phase shift revealed a significant main effect for Pendulum Combination (*F*(2, 25) = 40.378, *p* < 0.0001, $\eta_{p}^{2}=0.764$). In line with previous research, these results indicate greater phase shift for LEADER_FOLLOWER and FOLLOWER_LEADER compared to FOLLOWER_FOLLOWER (*p* < 0.05), respectively. As expected, *post hoc* comparisons revealed that phase shift values for both the relative and healthy control groups were significantly negative for LEADER_FOLLOWER (Relatives: *t*(26) = −4.807, *p* < 0.001; Controls: *t*(26) = −3.237, *p* < 0.005) and significantly positive for FOLLOWER_LEADER (Relatives: *t*(26) = 2.075, *p* = 0.048; Controls: *t*(26) = 2.096, *p* = 0.046) but not significantly different from zero for FOLLOWER_FOLLOWER (both *p* > 0.5). This analysis did not show any other significant effects (all *p* > 0.05); specifically, it did not show any effect of Group.

#### Circular variance of the relative phasing

The ANOVA on the circular variance of the relative phasing revealed significant main effects for Pattern (*F*(1, 25) = 25.480, *p* < 0.0001, $\eta_{p}^{2}=0.495$) and Pendulum Combination (*F*(2, 25) = 3.719, *p* = 0.039, $\eta_{p}^{2}=0.229$), indicating in line with previous research more stable coordination for the in-phase mode compared to the anti-phase mode and in the FOLLOWER_FOLLOWER compared to the LEADER_FOLLOWER and the FOLLOWER_LEADER pendulum conditions. Although the effect of group was non-significant (*F*(1, 25) = 1.774, *p* = 0.20, $\eta_{p}^{2}=0.06$), unaffected relatives were more variable in IIMC and when participants oscillated the same pendulum (FOLLOWER_FOLLOWER) compared to healthy controls.

To further explore this effect with the assumption that it might be moderated by attentional deficits of relatives, and thus, might be more likely to occur at the beginning of the trial, we divided each trial in two 30 s segments and used a 2 × 3 × 2 ANOVA with the factors Group (relatives, controls), Pendulum combination (LEADER_FOLLOWER, FOLLOWER_FOLLOWER, and FOLLOWER_LEADER), and Pattern (In-phase, Anti-phase) to examine the phase shift and the circular variance of the relative phasing of IIMC for first and second 30 s segments of the trials.

### Intentional interpersonal motor coordination analyzed in two segments

#### Phase shift of the relative phase

For both segments, the ANOVAs revealed a significant main effect for Pendulum Combination (First 30 s: *F*(2, 25) = 47.544, *p* < 0.001, $\eta_{p}^{2}=0.792$; Last 30 s: *F*(2, 25) = 46.926, *p* < 0.0001, $\eta_{p}^{2}=0.790$) and a significant interaction between Pattern and Pendulum Combination (First 30 s: *F*(2, 25) = 4.710, *p* < 0.005, $\eta_{p}^{2}=0.274$; Last 30 s: *F*(2, 25) = 6.116, *p* < 0.005, $\eta_{p}^{2}=0.329$) indicating, respectively, greater phase shift than FOLLOWER_FOLLOWER for LEADER_FOLLOWER and FOLLOWER_LEADER (*p* < 0.05) and a larger negative phase shift in anti-phase than in-phase for LEADER_FOLLOWER (*p* < 0.05). This pattern of results, like that for the entire trials reported above, replicates past findings (Amazeen et al., 1995).

**Figure 2:**
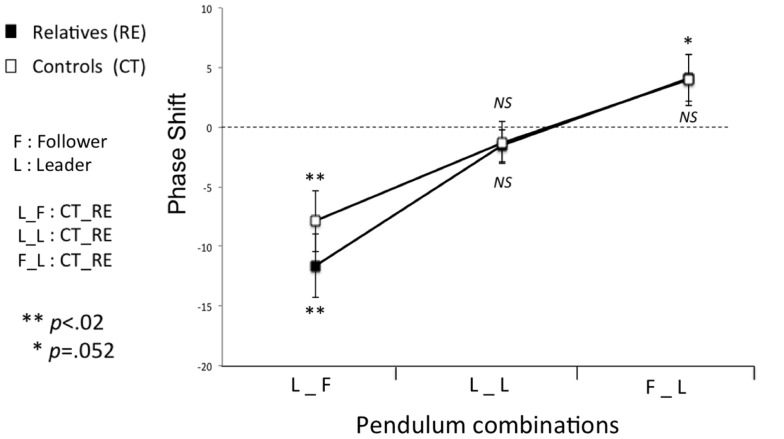
**The phase shift for intentional interpersonal motor coordination (First 30 s)**. Significantly different from zero for LEADER_FOLLOWER (*t*(26) = −4.561, *p* < 0.005) but not significantly different for FOLLOWER_FOLLOWER and FOLLOWER_LEADER (*p* > 0.05) in relatives group. Unaffected relatives never led the coordination. NS, not significant. Error bars represent standard error.

For the healthy control group, the pattern of single sample *t*-tests investigating Leadership was in line with predictions: significantly different from zero for LEADER_FOLLOWER and FOLLOWER_LEADER but not significantly different for FOLLOWER_FOLLOWER. These results indicate that the matched control subject, led when they had the smaller pendulum, followed when they had the larger pendulum and did not lead or lag when they had a pendulum of equal size to their partner. For the relatives group, however, these *t*-tests revealed a different pattern: significantly different from zero for LEADER_FOLLOWER for both the first and the second segment, significantly different from zero for FOLLOWER_LEADER for the First segment but not significant from zero for the second segment, and not significantly different for FOLLOWER_FOLLOWER for the first and the second segment (see Table 3). These results confirmed that, unaffected relatives did not lead the coordination during the first 30 s, even in those conditions in which they should have (fast pendulum LEADER) (see Figure 2).

**Table 3:** **Means ± SD of Leadership of participants in two segments**.

Pendulum combination	Relatives group (*n* = 27)	Zero	Statistics	*p*	$\eta_{p}^{2}$	Control group (*n* = 27)	Zero	Statistics	*p*	$\eta_{p}^{2}$
**FIRST SEGMENT OF 30 s**										
LEADER_FOLLOWER	−13.57 ± 13.62	0	*t* = −5.177	<0.001*	0.99	−9.34 ± 13.59	0	*t* = −3.573	<0.001	0.69
FOLLOWER_FOLLOWER	−2.23 ± 11.53	0	*t* = −1.008	0.323	0.19	−1.77 ± 6.74	0	*t* = −1.364	0.184	0.26
FOLLOWER_LEADER	5.69 ± 12.52	0	*t* = 2.362	0.026*	0.45	5.63 ± 7.82	0	*t* = 3.737	<0.001	0.72
**SECOND SEGMENT OF 30 s**										
LEADER_FOLLOWER	−11.63 ± 13.25	0	*t* = −4.561	<0.001*	0.87	−7.89 ± 13.76	0	*t* = −2.981	0.006	0.57
FOLLOWER_FOLLOWER	−1.56 ± 9.07	0	*t* = −0.894	0.379	0.17	−1.32 ± 7.07	0	*t* = −0.971	0.341	0.18
FOLLOWER_LEADER	4.12 ± 11.21	0	*t* = 1.756	0.156	0.33	3.96 ± 10.12	0	*t* = 2.036	0.052	0.40

^a^Single sample t-tests. *p < 0.05.

#### Circular variance of the relative phasing

**Figure 3:**
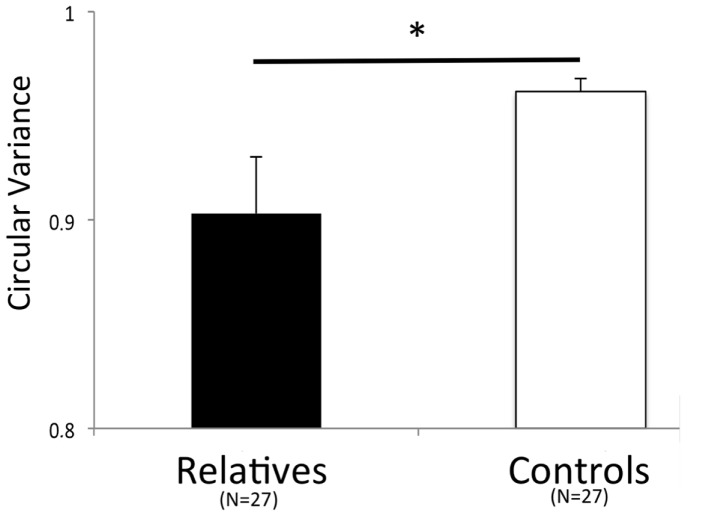
**Intentional interpersonal motor coordination variability (First 30 s)**. Significant difference between the two groups in anti-phase coordination (*F*(2, 25) = 4.279, *p* = 0.049, $\eta_{p}^{2}=0.141$). Error bars represent standard error.

The ANOVA performed on the circular variance values in intentional coordination task yielded a significant main effect of Pattern for both segments (First 30 s: *F*(2, 25) = 10.105, *p* < 0.005, $\eta_{p}^{2}=0.280$; Last 30 s: *F*(2, 25) = 14.983, *p* < 0.001, $\eta_{p}^{2}=0.366$). This result demonstrates that the in-phase pattern was more stable than the anti-phase pattern. We found a significant effect of Pendulum combination and a significant interaction between Pattern and Pendulum Combination only in the last 30 s of the trial (First 30 s: *F*(2, 25) = 1.523, *p* = 0.314; Last 30 s: *F*(2, 25) = 8.166, *p* = 0.002, $\eta_{p}^{2}=0.395$, and First 30 s: *F*(2, 25) = 1.213, *p* = 0.238; Last 30 s: *F*(2, 25) = 4.785, *p* = 0.017, $\eta_{p}^{2}=0.277$, respectively). These results demonstrate that swinging different pendulums with different eigenfrequencies induced instability as predicted by past research but only in the second half of the trials. However, the ANOVA also showed a significant main effect of Group only for the first 30 s of the trial (First 30 s: *F*(2, 25) = 4.279, *p* = 0.049, $\eta_{p}^{2}=0.141$; Last 30 s: *F*(2, 25) = 1.484, *p* = 0.234). Hence, these results indicate that the unaffected relatives were more variable in IIMC compared to healthy controls for the first half of the trials (see Figure 3).

### Correlations between cognitive variables, neurologic soft signs, and motor coordination performances

As motor behavior can be influenced by several potential confounding variables such as depression (psychomotor slowing, American Psychiatry Association, 2004) and anxiety level (anxiety affects the degree to which we (are able to) control our movements (Beilock and Gray, 2007), we controlled for these variables in correlational analyses. In addition, we used the NSS scale (Krebs et al., 2000), which is an indirect measure of motor behaviors abnormalities that might have been induced by cardiovascular diseases such as diabetic peripheral neuropathy (Bonnet and Lepeut, 2011) in correlational analyses. Results showed no significant correlations between cognitive (sustained attention), clinical (Neurologic soft sign) variables, and UIMC and IIMC performances (all *p*s > 0.05) in either the unaffected first-degree relatives or the control groups.

## Discussion

Identifying intermediate phenotypes for schizophrenia among at-risk samples has become a critical area of investigation that can possibly lead to more direct and earlier detection as well as intervention programs (Keshavan et al., 2008). IMC has been proposed as a potential candidate for intermediate phenotypes in schizophrenia (Varlet et al., 2012b). However, compared with the abundance of studies investigating cognitive intermediate phenotypes, research concerning motor coordination trait-markers, is in its early stage.

The present study evaluated two forms of IMC, IIMC, and UIMC, in relatives of patients compared to controls and examined the relationship between coordination performance, cognitive, and clinical variables. It replicated different results already in the literature. Unaffected individuals at family risk in our sample showed significantly lower visual sustained attention than healthy controls (Snizt et al., 2006). Concerning sensory and motor performance identified by clinical examination, relative’s participants presented significantly higher levels of NSSs compared to control participants (Chan et al., 2010; Neelam et al., 2011). But for the first time, controlling for potential confounding variables such as depression, NSSs, anxiety level, we have shown that IIMC, but not UIMC, is impaired in unaffected relatives of schizophrenia patients. In other words, relatives of patients have difficulties to voluntarily synchronize with a partner when they are explicitly asked to coordinate with. However, if no instructions were given, the relatives spontaneously synchronized with a partner in the same way than control participants. More specifically, employing a hand-held pendulum paradigm used by Varlet et al.’s (2012b) study on schizophrenia, we found that relative participants had greater variability and did not lead the coordination in the first part of the trial.

Previous research showed that the strength of the perceptual coupling, which influences the stability of such coordination, depends on how people visually pickup information about the movements of each other (Richardson et al., 2007). It has been demonstrated that a stronger perceptual coupling, and thus the more stable coordination, occurs when the movements of the other person are visually tracked with the eyes (Schmidt et al., 2007; Varlet et al., 2012a), or observed in central vision rather than peripheral vision (Richardson et al., 2007). These results are of particular interest in view of the impairment of the unaffected relatives group. Our study showed that unaffected participants had a sustained attention impairment compared to healthy controls. This result suggests that unaffected relatives may need more time to pickup information about the movements of other people. In addition, they may also need more time to implement efficient visual tracking. Several studies have shown that visual tracking was impaired in relatives of schizophrenia patients, characterized by global smooth pursuit dysfunction (Calkins et al., 2003, 2008).

Our findings also show that, similar to schizophrenia patients, their relatives did not lead the coordination even in the conditions in which they should have. A possible explanation of this result is that unaffected relatives not only have a weaker perceptual coupling but also have a time delay in processing information for visual motor coupling (Banerjee and Jirsa, 2007; Varlet et al., 2012a). Research on neuroanatomical connectivity might explain such a time delay. For example, in bimanual task, there is a contribution from the contralateral and ispsilateral motor cortices (Rokni et al., 2003). Thus, to move the right hand, information must be transmitted between both cortices. Callosal fibers allow interhemispheric crosstalk between the left and right hemispheres and several studies have elucidated their important role in creating temporal couplings (Kennerley et al., 2002). An anomaly in the callosal fibers could result in a slowdown in the transmission of interhemispheric information. Recently, Knöchel et al. (2012) has suggested that the volume of callosal fiber was significantly reduced in schizophrenia patients and in their relatives compared to healthy controls. Thus, in unaffected relatives, such as in schizophrenia, the reduction of volume of callosal fiber would lead to a reduction of transmission of interhemispheric crosstalk and thus to the occurrence of an offset in temporal coupling. Moreover, our results in the attentional processes task showed that relatives have reduced capacity for sustained attention compared to the control group. This observation strengthens the time delay explanation of the unaffected relatives’ phase shift. Thus, this offset in temporal coupling could result in the unaffected relatives not leading the coordination even in the conditions in which they should have. All these impairments (visual sustained attention, visual tracking and a time delay in processing information) might have contributed to an impaired social motor coordination in unaffected relatives.

Our results suggest that IIMC impairments could be consider as a potential intermediate phenotype of schizophrenia. Indeed, the results of our study are in line with the hypothesis that maintains that motor symptoms could constitute early symptoms of the schizophrenia spectrum. Importantly, the lack of correlation between clinical variables (i.e., depression, anxiety level, NSS) and IMC performances in relatives group suggests that our results are not due to these potential confounding variables. Past studies have found that neuromotor abnormalities are evident as early as infancy in prepsychotic individuals (Walker et al., 1994; Rosso et al., 2000), suggesting that movement abnormalities may also represent a core underlying vulnerability for psychosis and predict the eventual conversion to psychosis (Mittal and Walker, 2007). In this study, our results suggest that an IMC impairment matches the intermediate phenotype criteria for schizophrenia (Gottesman and Gould, 2003). As stated above, to be useful an intermediate phenotype must be: (1) associated with the illness; (2) heritable to some degree; (3) observed in unaffected family members to a greater extent than the general population; and (4) state independent in affected individuals (Gottesman and Gould, 2003). First, we know that IMC impairment is associated with schizophrenia (Varlet et al., 2012b). Secondly, our findings indicate that this impairment is heritable to some degree because we find it among unaffected parents of schizophrenia patients. Third, we show that IMC impairment is observed in unaffected family members to a greater extent than the general population. Unfortunately, the cross-sectional design of our study does not allow us to conclude for the fourth criterion “state independent in affected individuals,” (i.e., manifests in an individual whether or not illness is active). But, several studies suggest that adolescents who later develop schizophrenia exhibit motor behavior disorders, particularly motor dyscoordination, well before overt manifestations of the illness (Leask et al., 2002; Mittal et al., 2011). Future research should assess the presence of IMC impairment in adolescents with a high risk of schizophrenia in order to confirm the fourth criterion.

As integrative modeling of multivariate data from familial, neurobiological, socio-environmental, cognitive, and clinical domains represents a powerful approach to prediction of psychosis development (Shah et al., 2012), our results suggest that IIMC could be used as complementary measure to the existing ones in the early diagnosis of individuals with high risk of developing schizophrenia. However, further studies are needed to assess whether motor coordination could be a more sensitive measure or a more direct predictor of conversion than the existing ones. In addition, from a therapeutic point of view, recent data suggest the possibility that early psychosocial interventions with high-risk individuals might have the potential to delay the course or prevent the transition to psychosis (Stafford et al., 2013). Given that the effectiveness of an interaction has been shown to be directly linked to the degree of interpersonal coordination (Marsh et al., 2009), behavioral rehabilitation protocols that can help patients with schizophrenia to learn or relearn how to coordinate with the others might help patients adopt better natural social dynamics and better manage interpersonal relations in general.

The present study possesses some limitations. First, the sample size for the relatives group was small, which may have influenced the power of the tests to detect differences between the relatives group and the control group. Future research should confirm our findings using a larger sample. Another limitation is that the observation that the motor coordination impairments of schizophrenic patients (Varlet et al., 2012b) are found in their relatives may not be an argument for “genetic heritability.” Our results allow only the identification of so-called intermediate phenotypes that are detectable both in schizophrenia patients and in a higher proportion of their unaffected relatives than in the population at large. Moreover, future research should also confirm our findings in siblings of schizophrenia patients. Finally, due the cross-sectional design of our study, longitudinal studies are needed to objectively assess if motor coordination impairments could be precursor of psychosis.

## Conclusion

The main aim of the present study was to assess IMC in unaffected relatives of schizophrenia patients. For the first time, we showed that, whereas relatives of schizophrenia patients have normal UIMC, they have a lower stability of IIMC and did not lead the coordination in IIMC. These results have previously been found in schizophrenia patients; and consequently, one can infer that these social motor coordination impairments are associated with schizophrenia and that they are observed in unaffected relatives to a greater extent than the general population. Consequently, we have provided evidence to support the claim that social motor coordination could be considered as a trait-marker that might be associated with the risk for schizophrenia. This study showed the necessity to assess the motor dimension as an embodiment of mental disorders and to determine the impact of such impairments in daily living of patients and their relatives. The clinical significance of our finding lies in early diagnostics and in the field of preventative interventions, which therapeutically might target the motor aspect of social abilities in non-clinical individuals with a genetic risk of schizophrenia. Finally, future research should compare IMC with other mental disorders such as bipolar and autistic disorders, for example, in order to evaluate whether motor coordination impairment is a trait-marker that might be associated with social disorders in general.

## Authors Contributions

Jonathan Del-Monte, Stéphane Raffard, Delphine Capdevielle, Ludovic Marin, Robin N. Salesse, Richard C. Schmidt, Manuel Varlet, Benoît G. Bardy, Jean Philippe Boulenger, and Marie Christine Gély-Nargeot contributed to the study design. Stéphane Raffard recruited and Jonathan Del-Monte assessed the participants. Jonathan Del-Monte, Richard C. Schmidt, and Manuel Varlet performed the statistical analysis. Jonathan Del-Monte, Stéphane Raffard, Delphine Capdevielle, Ludovic Marin, and Manuel Varlet prepared the manuscript, with feedback from the other authors.

## Conflict of Interest Statement

The authors declare that the research was conducted in the absence of any commercial or financial relationships that could be construed as a potential conflict of interest.
